# The origin of parental care in relation to male and female life history

**DOI:** 10.1002/ece3.493

**Published:** 2013-02-20

**Authors:** Hope Klug, Michael B Bonsall, Suzanne H Alonzo

**Affiliations:** 1 Department of Biological & Environmental Sciences, University of Tennessee–ChattanoogaDept. 2653, 615 McCallie Aven, Chattanooga, Tennessee, 37403; 2 Department of Ecology & Evolutionary Biology, Yale UniversityPO Box 208106, 165 Prospect St, New Haven, Connecticut, 06520; 3 Mathematical Ecology Research Group, Department of Zoology, University of OxfordOxford, OX1 3PS, UK

**Keywords:** Biparental care, invasion analysis, life-history, maternal care, parental care, paternal care

## Abstract

The evolution of maternal, paternal, and bi-parental care has been the focus of a great deal of research. Males and females vary in basic life-history characteristics (e.g., stage-specific mortality, maturation) in ways that are unrelated to parental investment. Surprisingly, few studies have examined the effect of this variation in male and female life history on the evolution of care. Here, we use a theoretical approach to determine the sex-specific life-history characteristics that give rise to the origin of paternal, maternal, or bi-parental care from an ancestral state of no care. Females initially invest more into each egg than males. Despite this inherent difference between the sexes, paternal, maternal, and bi-parental care are equally likely when males and females are otherwise similar. Thus, sex differences in initial zygotic investment do not explain the origin of one pattern of care over another. However, sex differences in adult mortality, egg maturation rate, and juvenile survival affect the pattern of care that will be most likely to evolve. Maternal care is more likely if female adult mortality is high, whereas paternal care is more likely if male adult mortality is high. These findings suggest that basic life-history differences between the sexes can alone explain the origin of maternal, paternal, and bi-parental care. As a result, the influence of life-history characteristics should be considered as a baseline scenario in studies examining the origin of care.

## Introduction

Patterns of post-fertilization parental care are incredibly diverse (Clutton-Brock 1991). First, there is large disparity across taxa in whether any post-fertilization parental care is provided (Clutton-Brock 1991; Beck 1998; Reynolds et al. 2002). In birds, crocodiles, mammals, and cichlid fishes, one or both parents tend to care for young (Reynolds et al. 2002). In contrast, most non-cichlid teleosts, anurans, and squamate reptiles provide no care (Reynolds et al. 2002). Second, when care is provided, there is striking variation in which sex provides care (Clutton-Brock 1991; Beck 1998; Reynolds et al. 2002; Kokko and Jennions 2008). Bi-parental care is the norm in birds (Tullberg et al. 2002), maternal care is most common in mammals and invertebrates (Tallamy 1984, 2000; Zeh and Smith 1985; Clutton-Brock 1991), and paternal care is widespread in fishes that exhibit care (Blumer 1979; Reynolds et al. 2002; Mank et al. 2005). In amphibians, care is provided by either sex (Reynolds et al. 2002), whereas in reptiles, care tends to be maternal or bi-parental (Reynolds et al. 2002). Explaining such diversity has been the focus of extensive empirical and theoretical work (Blumer 1979; Baylis 1981; Tallamy 1984, 2000; Zeh and Smith 1985; Clutton-Brock 1991; Winemiller and Rose 1992; Beck 1998; Reynolds et al. 2002; Tullberg et al. 2002; Mank et al. 2005; Klug and Bonsall 2007, 2010; Kokko and Jennions 2008; Bonsall and Klug 2011a,b).

Classic theory suggests that females are more likely to provide parental care than are males, as they invest disproportionately more in gametes or zygotes (Trivers 1972), which decreases residual reproductive value and makes it beneficial for them to invest more heavily into current versus future reproduction (Sargent and Gross 1985; Coleman and Gross 1991; Gross 2005). Others have noted that past investment alone is insufficient to lead to sex differences in care (Dawkins and Carlisle 1976; Kokko and Jennions 2008) and have suggested a role for other factors in promoting differences between the sexes in parental investment. For instance, uncertainty of paternity is expected to make males more likely than females to abandon young on a macro-evolutionary scale (Trivers 1972). Within a species, uncertain paternity is predicted to affect paternal care if current and future reproductive opportunities vary in expected paternity (Trivers 1972; Baylis 1981; Westneat and Sherman 1993; Sheldon 2002; Alonzo 2010). Queller (1997) and Kokko and Jennions (2008) have shown that care by one sex can affect the availability of sexual partners and reproductive opportunities for the non-caring sex. As a result, sex ratios influence the fitness costs and benefits of caring versus deserting, which in turn determine whether males and/or females will be more likely to provide care (Queller 1997; Webb et al. 1999; Kokko and Jennions 2008). Furthermore, recent work has illustrated that the trade-off between current parental care and future mating success might not be as ubiquitous as previously assumed (Stiver and Alonzo 2009). In particular, if females prefer males that provide parental care, sexual selection is expected to favor male care (Alonzo 2012).

This theoretical work has led to considerable advances in our understanding of the evolution of care. Despite this, we are still far from understanding sex differences in parental care. Unexpected patterns of parental investment are the norm, and *post hoc* explanations, rather than well-supported *a priori* predictions, prevail in the literature (reviewed in Alonzo 2010). Previous theoretical work has tended to focus on the role of sexual selection in explaining sex differences in parental care. In contrast, relatively little work has explored how very basic and general life-history differences between males and females affect the evolution of care. A more comprehensive understanding of the evolution of parental care necessitates a closer look at how basic life history (i.e., stage-specific mortality, maturation rates) of males and females influences the evolution of maternal, paternal, and bi-parental care from an ancestral state of no care.

Within a species, males and females vary in numerous ways. Females initially invest more into zygotes than males. Additionally, one sex often has higher mortality during one or more life-history stages due to factors unrelated to parental investment, such as sex differences in physiology, mating behavior, predation risk, and resource use. Likewise, males and females often mature at different rates. Recent work even indicates that sex differences in life-history characteristics can arise during the egg stage in relation to yolk androgens (Sockman and Schwabl 2000; Eising et al. 2001; reviewed in Navar and Mendonça 2008). How such sex differences in life history influence the potential for maternal, paternal, or bi-parental care to originate is unknown. Our previous work suggests that life history can strongly influence the likelihood that some pattern of parental care will invade an ancestral state of no care (Klug and Bonsall 2010; Bonsall and Klug 2011a,b). Additionally, providing parental care is associated with costs and benefits, and such costs and benefits of care directly affect life-history traits (e.g., adult and offspring mortality). Thus, it is likely that sex-specific life-history characteristics will influence the origin of care.

Understanding how life history affects sex differences in parental care requires that we address two questions. First, how do male and female life-history characteristics affect the origin of some pattern of parental care from an ancestral state of no care, and second, once some pattern of care is present, how do male and female life-history characteristics influence transitions among paternal, maternal, and bi-parental care? In evolutionary models, it is important to distinguish between the origin and maintenance of parental care (Klug et al. 2012): in species with parental care, coevolution among traits (e.g., egg size and care) is expected to occur and individuals providing care typically experience higher mortality and/or lower current or future reproductive success; in species without parental care, such costs and potential for coevolution are absent. As a result, the dynamics that affect the origin and maintenance of care are expected to differ. Furthermore, independently examining the origin of and transitions among care allows for clear testable predictions that can be evaluated in a comparative or phylogenetic context. Thus, in this study, we focus only on the relationship between male and female life history and the origin of care. In related theoretical work (Klug et al. 2012), we examine transitions among different patterns of care.

Specifically, we examine the relationship between male and female basic life history (stage-specific mortality, rates of maturation) and the origin of maternal, paternal, and bi-parental care. We first identify combinations of male and female life-history characteristics that are most likely to lead to the origin of maternal, paternal, and bi-parental care from an ancestral state of no care. We then consider cases in which males and females vary in stage-specific mortality or maturation and ask which pattern of care will be most strongly favored from an ancestral state of no care based on these life-history differences.

## Methods

Using a mathematical model, we allow a rare mutant that exhibits paternal, maternal, or bi-parental care to invade a resident population in which care is absent (Metz et al. 1992; Dieckmann and Law 1996; Vincent and Brown 2005; Otto and Day 2007). The resident strategy is assumed to be in a stable equilibrium and the alternative parental care strategy invades from rare into the population (as is standard in invasion analyses; Otto and Day 2007). Building upon previous work (described in Klug and Bonsall 2007, 2010 and Bonsall and Klug 2011a,b), we assume a stage-structured system in which individuals pass through egg and juvenile stages and then mature and reproduce as adults. Mutant and resident individuals experience the same baseline conditions (i.e., both resident and mutant have the same death, maturation, and reproductive rates when no care is provided). Parental care is then assumed to be associated with benefits to offspring (increased survival beyond the baseline survival rate in the absence of care) and costs to the parent providing it (decreased parental survival relative to the no-care scenario; described below). In other words, we identify particular life-history parameters associated with a resident strategy of no care, and we then introduce a mutant that either exhibits paternal, maternal, or bi-parental care. We then evaluate the life-history parameters that will most strongly favor invasion by the mutant strategy.

Our approach differs from previous models in a number of key ways. First, we explicitly focus on all life-history stages (egg, juvenile, and adult) and ask how sex-specific life history can influence patterns of care. In contrast, many recent models on care focus on a single life-history stage (e.g., some models explore how differences between male and female adults can affect care). Second, we assume that females are the limiting sex (described below), but beyond that, we do not explicitly focus on how sex differences in mate competition influence parental care, a major focus in many recent models of parental care. As we focus explicitly on how life-history differences can drive patterns of care and focus minimally on sexual selection, our modeling framework can potentially serve as a null or baseline scenario for models that explore more detailed dynamics related to sex differences in mate competition and sexual selection.

### Model Dynamics

Males and females pass through egg (*E*) and juvenile stages and mature and reproduce as adults (*A*). Eggs decrease as they die and mature and increase as adults reproduce, such that


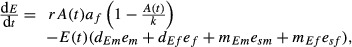
(1)

where *e*_*m*_ is the rate at which male eggs are produced and *e*_*f*_ is the rate at which female eggs are produced at time *t* (*e*_*m*_ = *e*_*f*_ = 0.5 initially in all cases considered). Male and female eggs die at rates *d*_*Em*_ and *d*_*Ef*_. At any given time, the rate of male eggs surviving the egg stage, *e*_*sm*_, equals *e*_*m*_(1 − *d*_*Em*_). Likewise, the rate of female eggs surviving the egg stage, *e*_*sf*_, equals *e*_*f*_(1 − *d*_*Ef*_). Those surviving male and female eggs then mature at rates *m*_*Em*_ and *m*_*Ef*_. Female fecundity limits reproduction (Bateman 1948) and reproduction in the population is assumed to be density-dependent. On average, each female produces *r* eggs that are fertilized. The total number of eggs that are fertilized is a function of *r*, the number of adults present *A(t)*, the rate at which females enter the adult stage *a*_*f*_, and the carrying capacity of the population *K*. The rate at which females enter the adult stage at time *t*, *a*_*f*_, equals *e*_*f*_(1 − *d*_*Ef*_)*m*_*Ef*_*σ*_*Jf*_, where *σ*_*Jf*_ represents female juvenile survival. Each fertilized egg has one mother and one father, and thus our measure of per capita female fecundity, *r*, is also a measure of the rate of egg fertilization in the population.

Adults in the population increase as individuals pass through the juvenile stage and decrease as adults die:


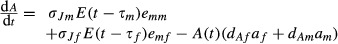
(2)

where *σ*_*Jm*_ and *σ*_*Jf*_ represent the juvenile survival rates of males and females, *e*_*mm*_ *and e*_*mf*_ are the rate of male and female eggs surviving the egg stage and maturing into juveniles, *τ*_*m*_ and *τ*_*f*_ are the durations of the male and female juvenile stages, and *d*_*Am*_ and *d*_*Af*_ are the rates at which male and female adults die. The rate at which males and females that survive the egg stage and mature into juveniles at time *t*, *e*_*mm*_ *and e*_*mf,*_ equals *e*_*m*_(1 − *d*_*Em*_)*m*_*Em*_ and *e*_*f*_(1 − *d*_*Ef*_)*m*_*Ef*_. The adults that are male and female at time *t* is a function of the rate of individuals surviving the egg stage, maturing, and surviving and passing through the juvenile stage. Specifically, the rate at which males and females enter the adult stage at time *t*, *a*_*m*_ *and a*_*f,*_ equals *e*_*m*_(1 − *d*_*Em*_)*m*_*Em*_*σ*_*Jm*_ and *e*_*f*_(1 − *d*_*Ef*_)*m*_*Ef*_*σ*_*Jf*_.

The density of resident adults at equilibrium (i.e., when 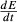 and 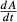 equal zero) is


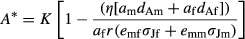
(3)

where *η* = *e*_*sf*_*m*_*Ef*_ + *e*_*sm*_*m*_*Em*_ + *e*_f_*d*_*Ef*_ + *e*_*m*_*d*_*Em*_.

The dynamics of the rare mutant that provides parental care are given by the following equations and by incorporating the relevant trade-offs associated with parental care into the mutant and resident dynamics (discussed below and in Table 1). The other parameters are as described previously and superscript denotes the new mutant strategy that exhibits parental care:


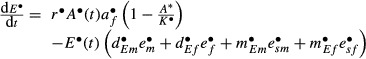
(4)


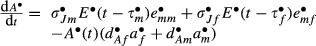
(5)

**Table 1:** Costs and benefits of initial investment in eggs by females (*1-d_Emo_*and *1-d_Efo_*) and parental care by males and females (*c*_*m*_ and *c*_*f*_). The total level of parental care provided to eggs, *c*_*total*_, is the sum of care provided by their mother and father, that is, *c*_*m*_ + *c*_*f*_. Male and female egg death rate decreases as initial investment in eggs increases and as the total level of parental care increases. Initial egg investment is costly to mothers, and female adult death rate increases and fecundity decreases as initial egg investment increases. Care is costly to parents, and as care increases, adult death rate also increases. These trade-offs are incorporated into the model and allow us to determine the fitness associated with various care scenarios (described in text). The term *a* determines the specific shape of the trade-off function and is equal to 6 in all cases considered

	Strategy:		
	
Life-history trait:	1. No care	2. Parental care of eggs	Example of trade-off:
Egg death rate (*d*_*Em*_ *& d*_*Ef*_)	*♂s*: 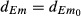 ♀s: 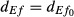	Egg death rate ↓ as care ↑ *♂s*: 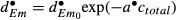 ♀s: 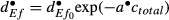	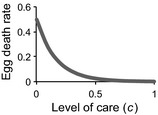
Adult death rate (*d*_*Am*_ *& d*_*Af*_)	Female adult death rate ↑ as initial egg investment ↑ *♂s*: 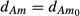 *♀s*: 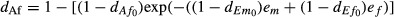	Male adult death rate ↑ as care ↑ and Female adult death rate ↑ as initial egg investment ↑ and as care ↑ *♂s*: 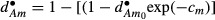 *♀s*: 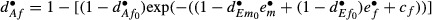	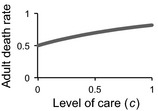
Female fecundity (*r*)	Female fecundity ↓ as initial egg investment ↑, i.e., Female fecundity ↓ as egg death rate in the absence of care ↓ 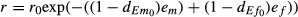	Female fecundity ↓ as initial egg investment ↑, that is, Female fecundity ↓ as egg death rate in the absence of care ↓ 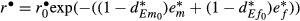	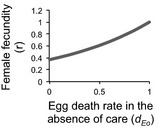

where A* (eqn. 3) is the equilibrial abundance of the resident adult population. As the mutant is assumed to be rare in the population, density-dependence operating on adult mutant reproduction occurs through competition with the resident (as is standard for ecological and evolutionary invasion analyses; e.g., Otto and Day 2007).

To explore the invasion of parental care from an ancestral state of no care, we consider the case in which rare adult mutants are present and able to provide parental care to their offspring, and pass on the gene(s) for a particular pattern of care to their offspring. This means that when we consider the case of paternal or maternal care, expression of the gene(s) for care is sex-limited and when we consider the case of bi-parental care, both sexes express the gene(s) for care. As parents are assumed to be able to provide care, we additionally assume that mutant parents are associated with their offspring during the parental care stage and remain alive long enough to provide care to young. Furthermore, the model assumes that at least a single male and single female of each strategy remain alive, and the parameter values considered never result in complete mortality of all of one sex. We do not specify how the mutant that provides a particular pattern of parental care arises in the population. The new mutant strategy could be the result of a genetic mutation within the population or immigration from another population. All offspring of mutant parents are assumed to exhibit the mutant strategy.

### Costs and benefits of parental care and initial egg investment

Parents can affect offspring survival and quality by investing resources into eggs (referred to herein as initial egg investment) and providing post-fertilization parental care behavior (referred to as parental care) to offspring (see also Klug and Bonsall 2010). Here, we assume that females initially allocate resources to eggs, and either male, female, or both male and female mutant parents can provide care to their eggs. For simplicity (and in line with our previous work – Klug and Bonsall 2007, 2010; Bonsall and Klug 2011a,b), we focus on parental care of developing zygotes and assume that juveniles do not receive care. This modeling framework is more generally representative of any system in which there are sequential development stages and parental care is provided only during the first stage.

Baseline egg death rate (i.e., egg death rate in the absence of any care) is used as our proxy of initial egg investment. By our definition, egg survival increases as initial egg investment increases. Initial egg investment is costly to females, such that as initial egg investment increases, female survival and fecundity decrease (Table 1). This assumes that an increase in individual egg size is associated with an increase in total investment within a given reproductive bout. Importantly, because this assumption is unchanged across all of our scenarios, this basic assumption is unlikely to affect our general patterns. Parental care, which again is provided by mutant parent(s) to their mutant eggs, increases egg survival, and the total level of care that eggs receive is the sum of the care provided by their male and female parents (*c*_*m*_ + *c*_*f*_) (Table 1). Providing care is costly to the parent providing it, and as the level of care increases, adult survival declines (i.e., male and/or female death rate increases) (Table 1). In the current model, we do not assume an explicit trade-off between parental care and female fecundity in order to keep the trade-offs between males and female as similar as possible. However, because a reduction in adult survival reduces future reproductive opportunities, there is also an indirect trade-off between care and future reproduction for both sexes. Minimizing baseline differences between the sexes in the costs of care allows us to determine whether sex-specific patterns of care arise because of sex differences in life-history characteristics (i.e., different mortality and maturation rates) or because females, by definition, initially invest more into eggs than male. Additionally, the assumption that female fecundity declines as females initially invest more into eggs does not affect the qualitative patterns – that is, if this trade-off is removed, the patterns are qualitatively similar.

In all cases, we assume that mutant adult parents are able to provide care for their young. This means that adult mutants either live long enough such that they are able to provide some level of care or that the benefits of care are present after their death (e.g., as would occur in matrophagy, which is common in some spiders). Likewise, we assume that mutant males and/or females are physically capable of providing care, and that when care is uni-parental, the expression of the gene(s) for care is sex-limited. We also assume that males and females have the potential to provide equivalent levels of care (Table 1). While this might not apply in all cases, we believe that this is the most biologically plausible assumption for early in the evolution of care and is a reasonable baseline scenario to consider.

In all cases, we assume asymptotic non-linear trade-offs (Table 1) because they allow for a broad exploration of parameter space, and hence, a thorough exploration of the costs and benefits of care. Specifically, using non-linear, asymptotic trade-off functions (Table 1) allows us to consider all biologically realistic parameter values (e.g., death and maturation rates that give rise to mortality that is between zero and one). In contrast, if we used linear trade-offs, we would only be able to consider a truncated range of parameter space (i.e., only those parameter values which gave rise to biologically sensible death and maturation). Non-linear trade-off functions are likely to be biologically realistic in many animals (Clutton-Brock 1991), and our general patterns will hold for other similarly shaped functions.

The trade-offs described in Table 1 provide some insight into the potential for parental care to increase reproductive success. However, the costs and benefits associated with care alone do not provide information on whether parental care will be able to invade a resident strategy of no care and persist given the stage-structured life-history conditions and the ecological dynamics. Information on invasion of care necessitates further analysis and is described below. These invasion analyses allow us to ask whether paternal, maternal, and/or bi-parental care can invade an ancestral state of no care given a set of specified male and female life-history parameters. This, in turn, allows us to identify the male and female life-history characteristics (stage-specific mortality and maturation) that are most likely to favor the origin of paternal, maternal, and/or bi-parental care.

### Fitness of parental care & invasion dynamics

The fitness of the rare mutant is a Fisherian measure of fitness and is expressed in terms of the per capita population-level growth rate of the rare mutant. More specifically, this fitness measure is determined from an invasion matrix (in which the entries in this matrix are the linearized mutant dynamics when the resident strategy is at equilibrium). Per capita growth rate is then the dominant eigenvalue associated with this invasion matrix and the fitness of the mutant that provides parental care is found by taking the determinant of:


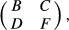
(6)

where


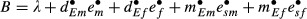
(7)


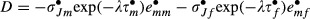
(8)


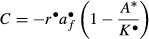
(9)


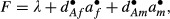
(10)

and solving the resulting characteristic equation for λ (i.e., the fitness of the mutant strategy relative to that of the resident; see also Metz et al. 1992 and Vincent and Brown 2005) when selection is relatively weak (*λ* is small such that exp(−*λτ*) ≈ (1 − *λτ*)). When λ is positive, the mutant strategy is predicted to invade the population; when λ is negative, the resident strategy of no care will persist in the population. Examining the relationship between λ and life-history traits of interest (mortality and maturation rates) provides insight into the qualitative relationship between those traits and the fitness associated with paternal, maternal, and bi-parental care. This allows us to determine when a particular pattern of care will be most strongly favored for a given set of life-history characteristics. Previous analyses of this general framework (Klug and Bonsall 2007) have demonstrated that the invasion dynamics are stable under the parameter range considered (see Fig. legends for details of parameter values considered).

In all cases, we assume that baseline conditions are identical for the mutant and resident strategy. We then calculate the fitness of the mutant strategy (paternal, maternal, or bi-parental care) relative to that of the resident strategy of no care in relation to varying male and female life-history parameters. In doing so, we focus on the evolutionary origin of some pattern of parental care. As mentioned above, this focus allows us to avoid confounding or even conflating the origin and maintenance of care, which are both interesting but distinct questions that involve differing evolutionary dynamics. We are interested in differences in the life-history conditions that favor the origin of paternal, maternal, and bi-parental care, and thus, we focus on cases in which care is beneficial and likely to be selected for. Our focus is on asking which pattern of care is predicted to evolve when parental care is favored by selection. Specifically, we consider parameter values in which care results in net benefits, and we then ask how male and female life-history characteristics influence the conditions under which each pattern of care is *most likely* to invade (i.e., the conditions under which a particular pattern of care is associated with the *greatest* fitness benefits) when care does increase offspring survival. In all analyses (Figs. 4), different numerical parameter values (e.g., higher or lower egg or adult mortality or maturation in a given analysis) will lead to different absolute fitness. However, it is the qualitative relationship between the life-history parameter considered and fitness that informs us of when selection will most strongly favor each pattern of care.

**Figure 1:**
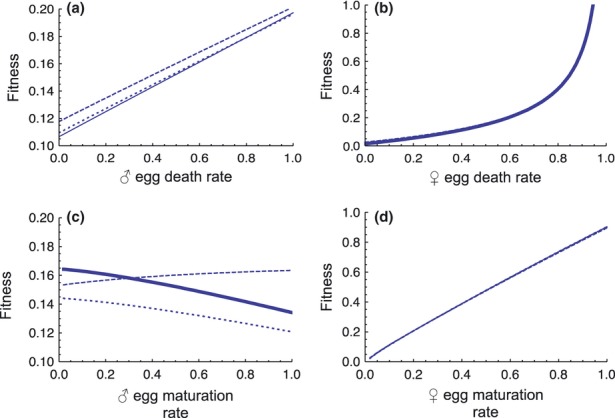
The origin of paternal (*c*_*m*_ = 0.7, *c*_*f*_ =0, solid line), maternal (*c*_*m*_ = 0.0, *c*_*f*_ =0.7, dashed line), and bi-parental (*c*_*m*_ = 0.35, *c*_*f*_ =0.35, dotted line) care is affected by life history associated with the egg stage. Here, we show the fitness gains associated with each pattern of parental care relative to the ancestral condition of no care for (A) male egg death rate in the absence of care, (B) female egg death rate in the absence of care, (C) male egg maturation rate, and (D) female egg maturation rate. All patterns of care will result in greater fitness benefits relative to the no-care scenario when male and female egg death rates are high (A-B) and when female eggs mature relatively quickly (D). Paternal and bi-parental care will be more likely to invade when male egg maturation rates are low, whereas maternal care is more likely to invade when male egg maturation rates are high (C). Unless otherwise noted, *d*_*Em0*_ = *d*_*Ef0*_ = 0.5, *m*_*Em*_ = *m*_*Ef*_ = 0.1, *r*_0_ =6, *d*_*Am0*_ = *d*_*Af0*_ = 0.5, *K* = 50, *σ*_*Jm0*_ = *σ*_*Jf0*_ = 0.5, *τ*_*m*_ = *τ*_*f*_ = 0.1, *e*_*m*_ = *e*_*f*_ = 0.5 for both residents and mutants. Note: a single line indicates that the fitness of paternal, maternal, and bi-parental care are indistinguishable and the individual lines overlap.

**Figure 2:**
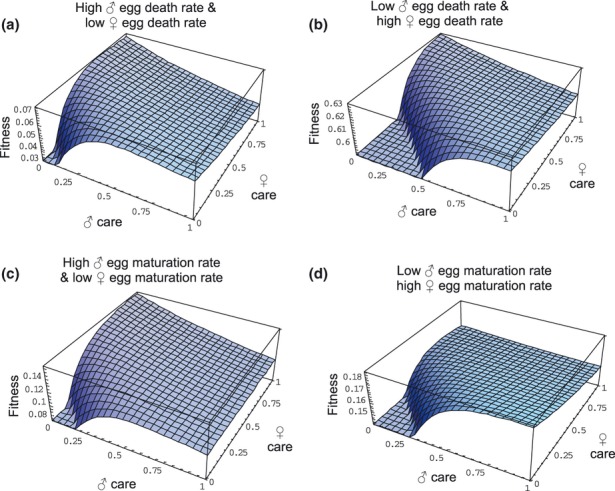
Difference in egg characteristics between the sexes favor maternal or paternal care. Here, we show fitness associated with the level of male and female care when (A) male egg death is high (0.9) and female egg death rate is low (0.1), (B) male egg death rate is low (0.1) and female egg death rate is high (0.9), (C) male egg maturation rate is high (0.9) and female egg maturation rate is low (0.1), and (D) male egg maturation rate is low (0.1) and female egg maturation rate is high (0.9). Maternal care will be more likely to invade when male eggs mature fast in comparison with female eggs (C). In contrast, paternal care will be more likely to invade when female eggs mature faster than male eggs (D). Unless otherwise noted, *d*_*Em0*_ = *d*_*Ef0*_ = 0.5, *m*_*Em*_ = *m*_*Ef*_ = 0.1, *r*_*0*_ =6, *d*_*Am0*_ = *d*_*Af0*_ = 0.5, *K* = 50, *σ*_*Jm0*_ = *σ*_*Jf0*_ = 0.5, *τ*_*m*_ = *τ*_*f*_ = 0.1, *e*_*m*_ = *e*_*f*_ = 0.5 for both residents and mutants.

**Figure 3:**
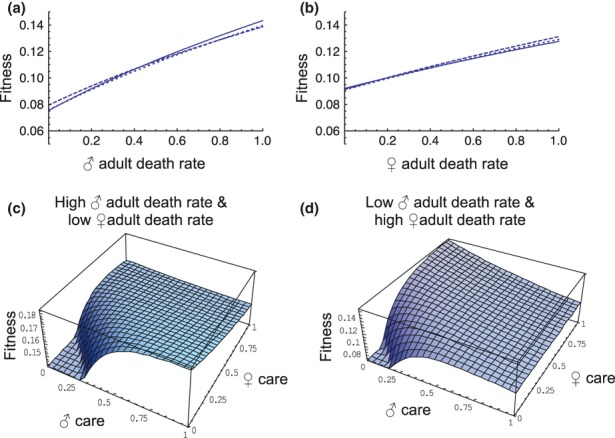
Adult mortality affects the origin of paternal (*c*_*m*_ = 0.7, *c*_*f*_ =0, solid line), maternal (*c*_*m*_ = 0.0, *c*_*f*_ =0.7, dashed line), and bi-parental (*c*_*m*_ = 0.35, *c*_*f*_ =0.35, dotted line) care. We show the fitness gains associated with each pattern of parental care relative to the ancestral condition of no parental care for (A) male adult death rate in the absence of care and (B) female adult death rate in the absence of care. We also show the fitness associated with different levels of male and female care when (C) male adult death rate is high (0.9) and female adult death rate is low (0.1), and (D) male adult death rate is low (0.1) and female adult death rate is high (0.9). Maternal, paternal, and bi-parental care will each be more likely to invade an ancestral state of no care when adult death rates are high (A-B). When male adult death rate is relatively high, paternal care will be most strongly favored (C), whereas when female adult death rate is relatively high, maternal care will be most likely to invade (D). Unless otherwise noted, *d*_*Em0*_ = *d*_*Ef0*_ = 0.5, *m*_*Em*_ =*m*_*Ef*_ = 0.1, *r*_0_ =6, *d*_*Am0*_ = *d*_*Af0*_ = 0.5, *K* = 50, *σ*_*Jm0*_ = *σ*_*Jf0*_ = 0.5, *τ*_*m*_ = *τ*_*f*_ = 0.1, *e*_*m*_ = *e*_*f*_ = 0.5 for both residents and mutants.

**Figure 4:**
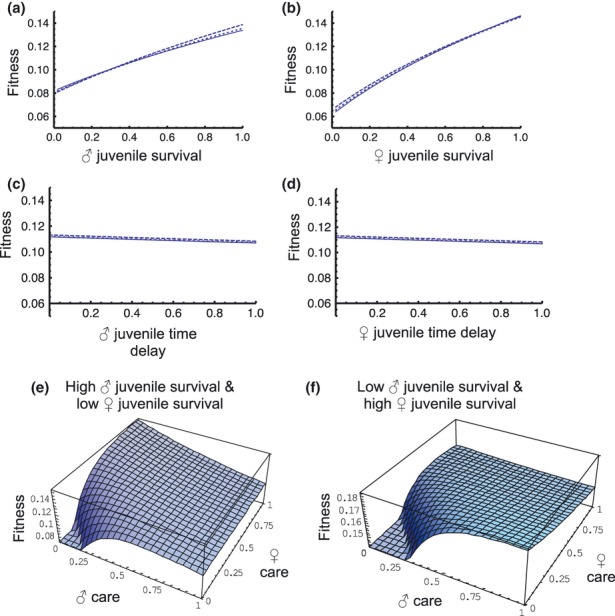
Juvenile traits affect the origin of paternal (*c*_*m*_ = 0.7, *c*_*f*_ =0, solid line), maternal (*c*_*m*_ = 0.0, *c*_*f*_ =0.7, dashed line), and bi-parental (*c*_*m*_ = 0.35, *c*_*f*_ =0.35, dotted line) care. We show the fitness gains associated with each pattern of parental care relative to the ancestral condition of no parental care for (A) male juvenile survival, (B) female juvenile survival, (C) male juvenile time delay, and (D) female juvenile time delay. We also show the fitness gains associated with various levels of male and female care relative to the no-care scenario when (E) male juvenile survival is greater than female juvenile survival (0.9 vs. 0.1) and (F) male juvenile survival is less than female juvenile survival (0.1 vs. 0.9). All patterns of care are more likely to invade an ancestral state of no care when juvenile survival is high (A-B). If male juvenile survival is relatively high, maternal care will be associated with the greatest fitness gains (E). If female juvenile survival is relatively high, paternal care will be most strongly favored (F). Unless otherwise noted, *d*_*Em0*_ = *d*_*Ef0*_ = 0.5, *m*_*Em*_ = *m*_*Ef*_ = 0.1, *r*_*0*_ =6, *d*_*Am0*_ = *d*_*Af0*_ = 0.5, *K* = 50, *σ*_*Jm0*_ = *σ*_*Jf0*_ = 0.5, *τ*_*m*_ = *τ*_*f*_ = 0.1, *e*_*m*_ = *e*_*f*_ = 0.5 for both residents and mutants.

In all cases, we first identify the relationship between the fitness benefits associated with paternal, maternal, and bi-parental care and male and female egg mortality, egg maturation rate, juvenile survival, duration of the juvenile period, and adult mortality. In doing so, we identify the life-history characteristics of males and females that will most strongly select for the origin of paternal, maternal, and bi-parental care from an ancestral state of no care. In many animals, males and females vary in life-history characteristics. Such differences between sexes likely affect the pattern of care that will occur. Thus, we also consider several scenarios in which males and females differ substantially in key life-history characteristics. We illustrate the dynamics using cases in which there are large differences between male and female egg mortality, egg maturation, juvenile survival, and adult mortality. However, the same general patterns are predicted for smaller qualitatively similar differences. For these scenarios, we then ask whether males and/or females will be most likely to provide parental care.

## Results

**Figure 5:**
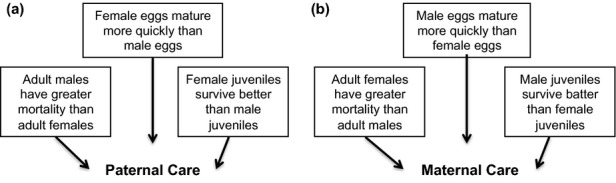
Differences between male and female life-history traits affect the origin of maternal and paternal care from an ancestral state of no care. (A) Paternal care will be most likely if female eggs mature relatively quickly, female juveniles have relatively high survival, and adult males have relatively high mortality. (B) In contrast, maternal care will be more likely if male eggs mature relatively quickly, male juveniles have relatively high survival, and adult females have relatively high mortality.

Male-only, female-only, and bi-parental care can originate over a wide range of male and female life-history traits (Fig. 5). Furthermore, there are little differences between the life-history conditions favoring each pattern of care from an ancestral state of no care – in other words, the life-history conditions that give rise to one pattern of care are similar to the conditions that give rise to other patterns of care (Table 2; Figs 1, 3, 4). States of paternal, maternal, and bi-parental care are most likely to evolve from a state of no care when egg death rate in the absence of care is high (Fig. 1A and B). In particular, some pattern of care will be strongly selected for if female egg death rate is high in the absence of care (Fig. 1B). Maternal care will result in slightly greater fitness gains than paternal or bi-parental care across the range of egg death rates (Fig. 1A). This remains true if male and female egg mortality varies substantially. When male egg death rate is very high and female egg death rate is very low, high levels of maternal care and little to no male care will result in the greatest fitness benefits to the mutants relative to fitness associated with no care (Fig. 2A). The qualitative pattern is identical when female egg death rate is much greater than male egg death rate (Fig. 2B).

**Table 2:** Life-history conditions that will most strongly favor paternal, maternal, and bi-parental care from an ancestral state of no care. There are few differences between the conditions that favor the origin of paternal, maternal, and bi-parental care. The exception is egg maturation rate of males: relatively slow male egg maturation rate is more likely to favor paternal or bi-parental care, whereas relatively fast-developing male eggs will favor the origin of maternal care

Type of parental care:		

Paternal	Maternal	Bi-parental
Low male egg maturation rates	High male egg maturation rates	Low male egg maturation rates

High female egg maturation rates		

High male and female egg death rates		

High male and female adult death rates		

High male and female juvenile survival		

Female egg maturation rate also has strong effects on the fitness associated with some pattern of care relative to the no-care scenario (Fig. 1D). If female eggs mature relatively quickly, maternal, paternal, and bi-parental care will be strongly favored (Fig. 1D). Male egg maturation rate has smaller effects on the fitness gains associated with care. If male eggs mature relatively slowly, paternal care will result in larger fitness gains than maternal care relative to the no-care scenario (Fig. 1C). If, however, male eggs mature relatively quickly, maternal care will result in the greatest fitness gains (Fig. 1C). When male and female egg maturation rates vary demonstrably, we see similar patterns. If male eggs mature much faster than female eggs, maternal care and little to no paternal care will be selected for (Fig. 2C). In contrast, if female eggs mature much faster than male eggs, high levels of paternal care and little to no maternal care will result in the greatest fitness gains to the mutants relative to that of the no-care resident strategy (Fig. 2D).

Baseline adult mortality also affects the fitness gains associated with parental care (Fig. 3). All patterns of care will result in the greatest fitness gains relative to the no-care scenario when both male and female adult death rates are high (Fig. 3A and B). This effect is consistent across the different care scenarios: the relationship between adult death rate and the fitness associated with care is similar regardless of whether care is maternal, paternal, or bi-parental (Fig. 3A and B). If, however, males and females have very different adult mortality, either maternal or paternal care will be selected for. Specifically, if male mortality is greater than female mortality, paternal care will result in the highest fitness gains relative to the no-care scenario (Fig. 3C). In contrast, if female mortality is much higher than male mortality, maternal care will be favored (Fig. 3D)

Parental care by either males and/or females is more likely to evolve when juvenile mortality is relatively low. The fitness benefit associated with care increases as either male or female juvenile survival increases, and this is equally true for the case of paternal, maternal, and bi-parental care (Fig. 4A and B). The duration of time spent in the juvenile stage has minimal effects on fitness associated with care, although all patterns of care result in slightly greater fitness gains when males and females spend more time as juveniles (Fig. 4C and D). If male juveniles have much greater mortality than female juveniles, maternal care will be most strongly favored. In contrast, if female juveniles have much greater mortality than males, paternal care will have the greatest fitness gains relative to the no-care scenario.

## Discussion

Male and female life-history characteristics affect the origin of paternal, maternal, and bi-parental care (Table 2; Fig. 5). In general, very similar life-history conditions favor the origin of paternal, maternal, or bi-parental care from an ancestral state of no care (Table 2). This means that, for example, the life-history conditions that are likely to favor maternal care are also likely to favor paternal and bi-parental care (and vice versa). Maternal, paternal, and bi-parental care are all most likely to originate from an ancestral state of no care when male and female egg death rates, adult mortality, and juvenile survival are high. When egg death rate in the absence of care is high, care often results in the greatest net benefits to offspring. In part, this occurs as egg survival (e.g., the proportion of eggs surviving per unit time) can never exceed one, and hence, when egg survival in the absence of care is already high, the benefit of care will be limited. The finding that care is favored when offspring need care the most is consistent with previous work (Stearns 1976; Clutton-Brock 1991; Webb et al. 2002; Klug and Bonsall 2010; Bonsall and Klug 2011a,b). Likewise, when baseline adult death rate (i.e., death rate in the absence of care) is high, care is often associated with smaller costs because adult mortality (i.e., the proportion of adults dying during any given time period) can also never exceed one. When adult death rate is high, parents also have reduced opportunity for future reproduction and are therefore expected to invest more heavily in current young. The finding that any pattern of care will be more likely when adult mortality is high is consistent with classic life-history theory (Stearns 1976) and previous empirical work that found a relationship between short lifespan and the evolution of parental care in fishes (Winemiller and Rose 1992).

All patterns of care will be favored from an ancestral state of no care when female egg maturation rate is high. Paternal and bi-parental care are slightly more likely when male eggs mature slowly, whereas maternal care is more likely when male eggs mature relatively quickly. Sex differences in egg maturation rate are not well studied. However, Cook and Monaghan (2003) found that in the back guillemot (*Cepphus grylle*), a species with bi-parental care, male chicks hatch on average 1 day sooner than female embryos. In the black-headed gull, Eising et al. (2001) found that injecting androgens into eggs resulted in faster hatching times. In contrast, Sockman and Schwabl (2000) found the opposite effect in American Kestrels. Development rate is affected by sex in other animals (Badyaev 2002), and recent work suggests that yolk androgens can lead to striking differences between the sexes in skeletal and neural development, immune function, and metabolic function even during the egg stage (reviewed in Navar and Mendonça 2008). As such differences arise between the sexes very early in development in some animals, it is possible that males and females begin to differ in survival and maturation as early as the egg stage. Regardless, the idea that offspring maturation rate can influence the evolution of sex-specific patterns of parental care is an intriguing possibility that warrants further attention.

The finding that a female's initial investment in eggs (i.e., egg death rate in the absence of care) does not substantially increase the likelihood of maternal care is contrary to some previous work. Classic and heavily influential theory suggests that females are more likely to care than males because females have greater initial investment in each offspring (Trivers 1972). This argument is only logical if the costs of greater initial female investment lead to reduced future expected reproductive success, which in turn selects for greater investment in current (rather than future) reproduction (Sargent and Gross 1985; see discussion of this hypothesis in Kokko and Jennions 2008). In our model, females always pay a greater cost of initially investing in eggs than males. However, the differences in fitness associated with maternal, paternal, and bi-parental care are similar across all values of initial maternal investment (i.e., baseline male and female egg death rate) in the early evolution of care (Fig. 2). Thus, in contrast to classic theory, we find that the origin of different patterns of care cannot be explained by initial differences between the sexes in gametic investment.

The fact that the origin of maternal, paternal, and bi-parental care cannot be explained by intersexual differences in residual reproductive value stemming from gametic or zygotic investment is consistent with recent parental investment theory. Mate competition and choice, trade-offs between time available to care versus time spent mating, and feedback between mate availability, care, and competition can favor one pattern of care over another (Queller 1997; Wade and Shuster 2002; Kokko and Jennions 2008; Alonzo 2010; Jennions and Kokko 2010). Interestingly, our findings suggest that even in the absence of such complexity (i.e., costs of mate competition, feedback between costs of competing or caring and mate availability), greater initial investment in zygotes is not sufficient to make maternal care more likely. Furthermore, the fact that maternal, paternal, and bi-parental care are favored when males and females are relatively similar suggests that explaining the origin of each pattern of care necessitates explicit consideration of life-history differences between males and females, costs of mate competition, and evolutionary feedback (Queller 1997; Kokko and Jennions 2008; Alonzo 2010). In other words, classic theory predicted that the prevalence of maternal care could be explained by sex differences in gametic investment between males and females. Recent work (Queller 1997; Kokko and Jennions 2008) and our findings show that this is not the case. Furthermore, the fact that we found that male and female care tends to be equally likely when males and females are relatively similar and when males are assumed to be the mate-limited sex illustrates that sex differences in life history and/or factors considered in previous models (Queller 1997; Jennions and Kokko 2008), including sex differences in the costs of mate competition or care and feedback among providing care, competing for mates, and mate availability are essential to explain sex differences in the origin of parental care. As sex-specific life-history differences can alone make one pattern of care more likely than another pattern of care, the effect of life-history differences must be considered as a baseline scenario in more complex models of care. In particular, our modeling framework provides a much-needed baseline scenario in which to examine conditions favoring the origin of parental care by males and/or females. This baseline scenario will allow for more explicit examination of the specific effects of complex dynamics in future models of care. In particular, an obvious next step is to examine the conditions that favor transitions among different patterns of care once some pattern of parental care is already present in a system (Klug et al. 2013).

When males and females differ in life-history characteristics for reasons unrelated to parental investment or care, paternal care will be most likely to evolve if male adult death rate is high relative to female adult death rate and if juvenile males mature slower and have higher mortality than females (Fig. 5A). Maternal care, on the other hand, will be most strongly favored if adult death rate is higher for females than males and if females mature slower and have greater juvenile mortality than males. The general finding that care will be more common in the sex with higher mortality is consistent with life-history theory suggesting that individuals with reduced expected future reproductive success should invest more in their current offspring (Williams 1966; Sargent and Gross 1985; Coleman and Gross 1991; Gross 2005; Klug and Bonsall 2010). These findings are also consistent with modeling by Steinhart et al. (2008) who found that adult survival is the most significant factor explaining whether parents abandon or care for their young in populations of smallmouth bass (*Micropterus dolomieu*). Likewise, Kokko and Jennions (2008) found that adult mortality will influence sex roles. However, in contrast to our findings, Kokko and Jennions (2008) found that the more common sex in the population (i.e., the sex with lower overall mortality) will typically be selected to provide more care than the rarer sex. This is because all offspring have one genetic mother and father, and as a sex becomes more common in the population, it becomes difficult to find a mate. In our model, each offspring has one mother and father because reproduction is limited by female fecundity. However, we do not assume a trade-off between caring and attaining mates (see Stiver and Alonzo 2009 for discussion of this issue). The differences between our findings and those of Kokko and Jennions (2008) suggest that whether such a trade-off is assumed, as well as the explicit consideration of mate competition and dynamic changes in which sex is mate-limited, can have important implications on the conditions that favor the origin of care by males and females.

In summary, male and female life history affects the origin of parental care. Paternal, maternal, and bi-parental care are most strongly favored by similar life-history conditions, although uni-parental care (maternal or paternal) is typically expected to arise from an ancestral state of no care in the early evolution of care. Differential investment between the sexes in gametes or zygotes cannot explain sex-specific patterns of care. In addition, sex-specific costs of mate competition and care and differential costs or benefits of care in relation to future mating success of the caring parent potentially influence life-history traits such as fecundity, rates of reproduction, male and female adult survival, and offspring survival. As a result, sex differences in the costs of caring and competing are expected to influence patterns of care (see, e.g., Baylis 1981; Queller 1997; Wade and Shuster 2002; Kokko and Jennions 2008; Alonzo 2010, 2012).
